# Supplementary material for: Intersectionality informed and narrative-shifting whole school approaches for LGBTQ+ secondary school student mental health: A UK qualitative study
Source: PLoS One. 2024 Jul 11;19(7):e0306864. doi: 10.1371/journal.pone.0306864 (PMC11238970; doi:10.1371/journal.pone.0306864)
Supplement: S1 Appendix — (DOCX) [file pone.0306864.s001.docx]

Table of Contents

[Student topic guide 1](#_Toc157513368)

[School staff topic guide 3](#_Toc157513369)

[Training provider topic guide 4](#_Toc157513370)

## Student topic guide

**Introduction**

- Thank participant for coming
- Reminder of aim of research and what it will involve: Interview (approx. 45 minutes) to learn about how school staff can help and support LGBT+ students at school or college through improving training provided
- Reminder of confidentiality and safeguarding:
  - Only the research team leads have access to your personal information provided in your consent form. The recording from today will be stored securely and will be deleted once we’ve written it up and removed any personally identifying information. Only break confidentiality if you tell us anything which we feel puts you or anyone else at any risk.
- Recheck consent to take part recording
  - No one will be able to link their answers back to them personally, including their school)– they will not get in trouble for any comments about their school
- Any questions?

**Questions**

1. **First of all, can you tell me a bit about what you think being at your school is like for LGBTQ+ students?**
2. **As we know, LGBTQ+ covers a broad range of young people - including those identifying as lesbian, gay, bisexual, trans, queer for example, but also those who have not come out or who are questioning – do you think there may be any differences in the ways in which they may experience school life?**
3. **From past research, we know that LGBTQ+ young people report greater difficulties with mental health and wellbeing, and more difficult experiences at school. Research also finds that those who share other marginalised identities such as LGBTQ+ young people who are Black, those who receive free school meals, or come from a faith background report greater mental health difficulties as well as additional challenges with school life. Do you have any thoughts about, or experiences of this at all?**

*Probe: How does this affect their mental health/wellbeing?*

***We know that some students experience bullying at school because other’s think they might be LGBTQ+ and some students report hearing negative language about sexual orientation or gender identity while at school…***

1. **How do you think staff at your school would respond if they overheard bullying or negativity towards LGBTQ+ students?**

*Probe: Why do you think they respond in this way?*

*Prompt: Can you think of anything that might stop staff stepping in in that situation, do you think?*

*Prompt: How would you want staff to respond?*

1. **How knowledgeable do you think school staff are about issues that affect LGBTQ+ students?**

*Probe: in what ways, if any, does your school support LGBTQ+ young people or discuss LGBTQ+ issues?*

*Prompt:* *Is there anything that you wish they did know or do differently?*

1. **What sorts of things do you think are important to include in training for school staff to support LGBTQ+ students?**
2. **Are there any ways that training be improved to ensure that all aspects of young people’s social identities are supported, including race and ethnicity, faith and socio-economic background?**
3. **Thinking about the different types of staff at your school - such as head teachers, other teachers, caretakers, people who clean, cook, and reception staff - what could they do to make the school a better environment for LGBTQ+ students?**

*Prompt: Are there any types of staff who may find it harder or less hard to discuss LGBTQ+ issues with students do you think? Why/why not?*

*Prompt: How do you think parents / carers would feel about school staff receiving training about LGBTQ+ issues?*

1. **Is there anything else you would want school staff to learn about or be better at doing to support LGBTQ+ students’ mental health, stress or wellbeing?**
2. **Do you think your school or staff at your school would face any challenges in taking up this type of training or putting their training into practice?**
3. **Is there anything else that you’d like to say about training for school staff to better support LGBTQ+ students?**
4. **We are coming to the end of the questions that I had, is there anything else that you thought we’d ask that we haven’t covered?**
5. **Finally, I just wanted to ask you how you’ve found today’s session?**

**Closing statement**

- Thank participant(s) for their time
- Draw attention to signposting sheet, let them know that they can get in touch if they would like to access support or want help in doing so
- Inform them about study next steps (analysing data and we will share findings) and how to collect voucher, let them know they can get back in touch if they would like to add anything
- Ask how they found out about the study and encourage to share with contacts/school if appropriate

## School staff topic guide

**Introduction**

- Thank participant for coming
- Reminder of aim of research and what it will involve – interview (approx. 45 minutes) to learn about how your school or college support young people who are or think they might be LGBT+, the skills/training you need to support their wellbeing and if there are any difficulties in accessing training or putting it into practice
- Recap key points of information sheet
  - Only research team will have access to personal information. It will be stored in a secure and password protected database. We will also store the information with a unique ID number rather than your name. Only break confidentiality if you tell us anything which we feel puts you or anyone else at any risk.
- Recheck consent to take part including recording (no one will be able to link their answers back to them personally, including their school)
- Any questions?

**Topic guide**

1. **Firstly then, can you describe in your own words how you think students who are, or think they might be, LGBTQ+ experience life at your school?**

*Probe: in what ways does the school environment affect them, in your experience?*

1. **As we know, LGBTQ+ covers a broad range of young people - including those identifying as lesbian, gay, bisexual or queer as well as trans or non-binary, for example, but also those who have not come out or who are questioning their identity. Do you think there may be any differences in how these different groups experience school life?**
2. **From past research, we know that LGBTQ+ young people report greater difficulties with mental health and wellbeing, and more difficult experiences at school compared to young people identifying as cis-gendered and/or heterosexual. Do you have any experiences of this at your school at all?**
3. **Research also finds that those who share other marginalised identities such as LGBTQ+ young people who are Black, those who receive free school meals, or come from any faith background report greater mental health difficulties as well as additional challenges with school life. Do you have any experiences of this at your school at all?**
4. **In what ways, if any, could staff (including teaching, domestic and administrative staff) support LGBTQ+ students at your school?**

*Probe: in what ways, if any, does your school support LGBTQ+ young people or discuss LGBTQ+ issues?*

*Probe: how might this be important for their mental health or wellbeing, if at all?*

Prompt: *are there any ways in which schools could be more aware of, or better support young people with multiple marginalised identities, do you think?*

1. **Do staff at your school receive any training to support LGBTQ+ students?**

***(If yes) Probe*** *- can you say more about what was included, what was good or could have been improved, if anything?*

***(if no) Probe*** *- can you say more about why you think they haven’t received any training in this area?*

1. **What types of skills, capacity or awareness do you think school staff need to support LGBTQ+ students in these ways?**

*Probe – are there any staff members who may find it harder or less hard to discuss LGBTQ+ issues with students?*

- *How could they be better supported?*

1. **Are there any ways that training be improved to ensure that all aspects of young people’s social identities are reflected in the training, including race and ethnicity, faith and/or socio-economic background?**
2. **Do you think your school or staff at your school would have any challenges in taking up training to support LGBTQ+ students or putting their training into practice?**

*Prompt: what could be done to overcome this, do you think?*

Probe: *(if not already covered) in your experience how do you think parents/carers would feel about school staff receiving this kind of training?*

*- Is that something schools need support with, in terms of liaising with parents/carers do you think?*

1. **Is there anything else that you’d like to say about training for school staff to better support LGBTQ+ students?**
2. **We are coming to the end of the questions that we had, is there anything else that you thought we’d ask that we haven’t covered?**
3. **Finally, I just wanted to ask you how you’ve found today’s session?**

**Closing statement**

- Thank participant(s) for their time
- Draw attention to signposting sheet, let them know that they can get in touch if they would like to access support or want help in doing so
- Inform them about study next steps (analysing data and we will share findings) and how to collect voucher, let them know they can get back in touch if they would like to add anything
- Encourage to share with contacts/school/students if appropriate

## Training provider topic guide

1. **First of all, can you say a bit about what role you think schools can play in supporting LGBTQ+ pupils’?**

- *What about their mental health?*

1. **Can you tell us more about the training you offer at schools?**

- *Probe about coverage of mental health and LGBTQ+ issues*
- *Who delivers this training?*
- *Are there any ways in which who provides the training makes any difference to how it is received or what impact it has do you think?*

1. **How was the training designed?**

- *Were young people involved? If so, how?*
- *Do you evaluate the training at all? If so, how?*

1. **Do you think the training you offer has an impact on pupil mental health?**
2. **What do you think are the most important components of schools training to best support LGBTQ+ students?**

- *What do you think staff find most helpful?*
- *What are the biggest gaps in their knowledge or skills in your experience?*
- *What do you think young people think is most important for school staff to know?*

1. **What is most important in terms of helping promote positive mental health or to prevent mental health problems among LGBTQ+ pupils?**
2. **Are there any types or groups of pupils who may find it harder to consider coming out at school do you think?**

- *Probe about racial and ethnic minority, religious or faith communities, socio-economically disadvantaged students?*
- *Do you touch on this in your training?*

1. **Why might staff not intervene they overheard bullying or negativity towards LGBTQ+ pupils?**

- *Do you touch on this in your training?*
- *Are there any groups or types of staff who would be more or less likely to intervene, do you think?*

1. **Thinking about the different types of school staff such as head teachers, other teachers, caretakers and people who clean, cook, administration staff and so on, what could they all do to make the school a better environment for LGBTQ+ students?**

- *Are there any things various types of staff could do differently to be more inclusive?*
- *Does your training provide targeted different types of staff?*

1. **What factors do you think affect whether or not schools take up training?**

- *What factors affect whether that training would be put into practice?*
- *Are there any ways that these barriers could be overcome?*

1. **Are there any other ways in which you think training for schools could be improved?**
2. **We are coming to the end of the questions that I had, I just wanted to ask you how you’ve found today’s session?**

- *Is there anything that you thought we would cover that you didn’t have a chance to say?*

[Thank participant(s) for their time]

[Draw attention to signposting sheet]

[Inform them about study next steps]
