# Supplementary material for: Intersectionality informed and narrative-shifting whole school approaches for LGBTQ+ secondary school student mental health: A UK qualitative study
Source: PLoS One. 2024 Jul 11;19(7):e0306864. doi: 10.1371/journal.pone.0306864 (PMC11238970; doi:10.1371/journal.pone.0306864)
Supplement: Attachment — Submitted filename: PONE-D-23-28894_reviewer.pdf [file pone.0306864.s002.pdf]

PLOS ONE

Intersectionality informed and narrative-shifting whole school approaches for LGBTQ+ secondary school student mental health: A UK qualitative study.

--Manuscript Draft--

**Manuscript Number:**	PONE-D-23-28894
**Article Type:**	Research Article
**Full Title:**	Intersectionality informed and narrative-shifting whole school approaches for LGBTQ+ secondary school student mental health: A UK qualitative study.
**Short Title:**	Intersectionality and LGBTQ+ secondary school inclusion
**Corresponding Author:**	Charlotte Woodhead<br>King's College London<br>London, UNITED KINGDOM
**Keywords:**	LGBTQ+; intersectionality; public mental health; secondary schools; gender and sexuality; qualitative
**Abstract:**	School is a key site for public mental health, with sexual and gender minority students being a priority group for action. Context is important in understanding how school inclusion of sexual and gender minorities shapes mental health and well-being, with rapidly changing social and political forces necessitating ongoing research. This coproduced UK secondary school-based study aimed to understand (a) key components of mentally, socially and emotionally healthy school environments for LGBTQ+ students considerate of intersecting minoritised identities; (b) staff information, skills and capacity needs and (c) factors influencing uptake and implementation. Online interviews and focus groups were conducted with 63 participants (22 staff, 32 students (aged 13-19 years), and 9 training providers), diverse in relation to gender and sexual identity, ethnicity, religious and social context. Data were analysed thematically. One overarching theme captured the need for an intersectionality-informed, contextually adaptable, whole school approach which 'shifts the narrative' away from deficit thinking, challenging prevailing cis/heteronormative and white norms. This underpinned four themes: (1) 'Feeling safe, seen and celebrated: Embedding intersectional signs, signals and symbols', (2) 'Everyone's business: the need for collaboration', (3) 'Embedding a culture of change', and (4) 'Re-locating the problem: challenging deficit thinking'. Contextually diverse research is needed which critically addresses ways in which social power enacted interpersonally and structurally serves to hinder schools from enacting LGBTQ+ inclusivity. Evidence to inform and develop implementation strategies for institutional changes and to advocate for wider socio-political support is also key to mitigate the potential for widening inequities linked to inequitable school environments.
**Order of Authors:**	Amy Morgan
	Emily Cunningham
	Juliet Dyrud
	Liberty Elliott
	Lauren Ige
	Gemma Knowles
	Lukasz Konieczka
	Angela Mascolo
	Ibrahim Sabra
	Sarah Sabra
	E Singh
	Katharine A Rimes
	Charlotte Woodhead

Powered by Editorial Manager® and ProduXion Manager® from Aries Systems Corporation

Additional Information:	
**Question**	**Response**
**Financial Disclosure**<br><br>Enter a financial disclosure statement that describes the sources of funding for the work included in this submission. Review the submission guidelines for detailed requirements. View published research articles from *PLOS ONE* for specific examples.<br><br>This statement is required for submission and **will appear in the published article** if the submission is accepted. Please make sure it is accurate.<br><br>**Unfunded studies**<br>Enter: *The author(s) received no specific funding for this work.*<br><br>**Funded studies**<br>Enter a statement with the following details:<br>• Initials of the authors who received each award<br>• Grant numbers awarded to each author<br>• The full name of each funder<br>• URL of each funder website<br>• Did the sponsors or funders play any role in the study design, data collection and analysis, decision to publish, or preparation of the manuscript?<br>• **NO** - Include this sentence at the end of your statement: *The funders had no role in study design, data collection and analysis, decision to publish, or preparation of the manuscript.*<br>• **YES** - Specify the role(s) played.<br><br>* typeset	This work was supported by the TRIUMPH (Transdisciplinary Research for the Improvement of Youth Mental Public Health) Network which is funded by the Cross-Disciplinary Mental Health Network Plus initiative supported by UKRI under grant ES/S004351/1. AM, CW, and GK are part supported by the ESRC Centre for Society and Mental Health at King's College London [ES/S012567/1]. The views expressed are those of the author(s) and not necessarily those of the ESRC or King's College London.
**Competing Interests**<br><br>Use the instructions below to enter a competing interest statement for this submission. On behalf of all authors, disclose any competing interests that could be perceived to bias this work—acknowledging all financial support and any other relevant financial or non-financial competing interests.	The authors have declared that no competing interests exist

Powered by Editorial Manager® and ProduXion Manager® from Aries Systems Corporation

This statement is required for submission and **will appear in the published article** if the submission is accepted. Please make sure it is accurate and that any funding sources listed in your Funding Information later in the submission form are also declared in your Financial Disclosure statement.<br><br>View published research articles from *PLOS ONE* for specific examples.<br><br>**NO authors have competing interests**<br><br>Enter: *The authors have declared that no competing interests exist.*<br><br>**Authors with competing interests**<br><br>Enter competing interest details beginning with this statement:<br><br>*I have read the journal's policy and the authors of this manuscript have the following competing interests: [insert competing interests here]*<br><br>* typeset	
**Ethics Statement**<br><br>Enter an ethics statement for this submission. This statement is required if the study involved:<br><br>• Human participants<br>• Human specimens or tissue<br>• Vertebrate animals or cephalopods<br>• Vertebrate embryos or tissues<br>• Field research<br><br>Write "N/A" if the submission does not require an ethics statement.<br><br>General guidance is provided below. Consult the submission guidelines for detailed instructions. **Make sure that all information entered here is included in the**	Both written and verbal consent was obtained from all participants. Ethical approval was received from King's College London Research Ethics Committee (Palliative care, Nursing and Midwifery Research Ethics Subcommittee), reference: HR-20/21-21515.

**Methods section of the manuscript.**<br><br>**Format for specific study types**<br><br>**Human Subject Research (involving human participants and/or tissue)**<br>• Give the name of the institutional review board or ethics committee that approved the study<br>• Include the approval number and/or a statement indicating approval of this research<br>• Indicate the form of consent obtained (written/oral) or the reason that consent was not obtained (e.g. the data were analyzed anonymously)<br><br>**Animal Research (involving vertebrate animals, embryos or tissues)**<br>• Provide the name of the Institutional Animal Care and Use Committee (IACUC) or other relevant ethics board that reviewed the study protocol, and indicate whether they approved this research or granted a formal waiver of ethical approval<br>• Include an approval number if one was obtained<br>• If the study involved *non-human primates*, add *additional details* about animal welfare and steps taken to ameliorate suffering<br>• If anesthesia, euthanasia, or any kind of animal sacrifice is part of the study, include briefly which substances and/or methods were applied<br><br>**Field Research**<br><br>Include the following details if this study involves the collection of plant, animal, or other materials from a natural setting:<br>• Field permit number<br>• Name of the institution or relevant body that granted permission	
**Data Availability**<br><br>Authors are required to make all data underlying the findings described fully available, without restriction, and from the time of publication. PLOS allows rare exceptions to address legal and ethical concerns. See the PLOS Data Policy and FAQ for detailed information.	No - some restrictions will apply

Powered by Editorial Manager® and ProduXion Manager® from Aries Systems Corporation

A Data Availability Statement describing where the data can be found is required at submission. Your answers to this question constitute the Data Availability Statement and **will be published in the article**, if accepted.<br><br>**Important:** Stating 'data available on request from the author' is not sufficient. If your data are only available upon request, select 'No' for the first question and explain your exceptional situation in the text box.<br><br>Do the authors confirm that all data underlying the findings described in their manuscript are fully available without restriction?	
**Describe where the data may be found in full sentences. If you are copying our sample text, replace any instances of XXX with the appropriate details.**<br><br>• If the data are **held or will be held in a public repository**, include URLs, accession numbers or DOIs. If this information will only be available after acceptance, indicate this by ticking the box below. For example: *All XXX files are available from the XXX database (accession number(s) XXX, XXX.).*<br>• If the data are all contained **within the manuscript and/or Supporting Information files**, enter the following: *All relevant data are within the manuscript and its Supporting Information files.*<br>• If neither of these applies but you are able to provide **details of access elsewhere**, with or without limitations, please do so. For example:<br><br>*Data cannot be shared publicly because of [XXX]. Data are available from the XXX Institutional Data Access / Ethics Committee (contact via XXX) for researchers who meet the criteria for access to confidential data.*<br><br>*The data underlying the results*	Data cannot be shared publicly because of the sensitive nature of the topic. Data are available from the King's College London Institutional Data Access (contact via King's Open Research Data System (KORDS) research.data@kcl.ac.uk) for researchers who meet the criteria for access to confidential data.

Powered by Editorial Manager® and ProduXion Manager® from Aries Systems Corporation

presented in the study are available from (include the name of the third party and contact information or URL).<br>• This text is appropriate if the data are owned by a third party and authors do not have permission to share the data.<br><br>* typeset	
Additional data availability information:	

Manuscript

Click here to access/download;Manuscript;PLoS ONE_LGBTQ+ student mental health 2.docx

Title: Intersectionality informed and narrative-shifting whole school approaches for LGBTQ+ secondary school student mental health: A UK qualitative study.

Short title: Intersectionality and LGBTQ+ secondary school inclusion

Amy Morgan[1,2], Emily Cunningham[3], Juliet Dyrud[1], Liberty Elliott[1], Lauren Ige[1], Gemma Knowles[1,4] https://orcid.org/0000-0001-6107-3957, Lukasz Konieczka[5], Angela Mascolo[1], Ibrahim Sabra[1], Sara Sabra[1], E. Singh[1], Katharine. A. Rimes[6], https://orcid.org/0000-0003-2634-455X Charlotte Woodhead[1,2,□] https://orcid.org/0000-0002-5545-6238

[1] ESRC Centre for Society and Mental Health, King's College London, Melbourne House, 44-46 Aldwych, London WC2B 4LL, United Kingdom

[2] King's College London, Department of Psychological Medicine, Institute of Psychiatry, Psychology & Neuroscience, 16 De Crespigny Park, London SE5 8AF, United Kingdom

[3] TRIUMPH Network; MRC/CSO Social and Public Health Sciences Unit, University of Glasgow

[4] King's College London, Department of Health Services and Population Research, Institute of Psychiatry, Psychology & Neuroscience, 16 De Crespigny Park, London SE5 8AF, United Kingdom

[5] Mosaic LGBT+ Young Person's Trust, London, United Kingdom

[6] King's College London, Department of Psychology, Institute of Psychiatry, Psychology & Neuroscience, 16 De Crespigny Park, London SE5 8AF, United Kingdom

[□]Corresponding author: Dr Charlotte Woodhead, ESRC Centre for Society and Mental Health, King's College London, Melbourne House, 44-46 Aldwych, London WC2B 4LL, United Kingdom; E-mail: charlotte.woodhead@kcl.ac.uk

Abstract

School is a key site for public mental health, with sexual and gender minority students being a priority group for action. Context is important in understanding how school inclusion of sexual and gender minorities shapes mental health and well-being, with rapidly changing social and political forces necessitating ongoing research. This coproduced UK secondary school-based study aimed to understand (a) key components of mentally, socially and emotionally healthy school environments for LGBTQ+ students considerate of intersecting minoritised identities; (b) staff information, skills and capacity needs and (c) factors influencing uptake and implementation. Online interviews and focus groups were conducted with 63 participants (22 staff, 32 students (aged 13-19 years), and 9 training providers), diverse in relation to gender and sexual identity, ethnicity, religious and social context. Data were analysed thematically. One overarching theme captured the need for an intersectionality-informed, contextually adaptable, whole school approach which ‘shifts the narrative’ away from deficit thinking, challenging prevailing cis/heteronormative and white norms. This underpinned four themes: (1) ‘Feeling safe, seen and celebrated: embedding intersectional signs, signals and symbols’, (2) ‘Everyone’s business: the need for collaboration’, (3) ‘Embedding a culture of change’, and (4) ‘Re-locating the problem: challenging deficit thinking’. Contextually diverse research is needed which critically addresses ways in which social power enacted interpersonally and structurally serves to hinder schools from enacting LGBTQ+ inclusivity. Evidence to inform and develop implementation strategies for institutional changes and to advocate for wider socio-political support is also key to mitigate the potential for widening inequities linked to inequitable school environments.

Introduction

LGBTQ+ young people (here defined as those “who identify as lesbian, gay, bisexual, transgender, and/or questioning, and/or who express diverse sexual orientations, gender identities, and/or gender expression” (Fisher et al., 2017; p.1) are a public mental health priority group (Taylor et al.,

2022). Stigma, discrimination, internalised homo/bi/transphobia, bullying, and victimisation are key predictors of mental distress for LGBTQ+ young people, alongside low self-esteem, lack of acceptance and support from parents and communities (Russell & Fish, 2016), poorer family relationships (Argyriou et al., 2020) and exposure to sexual, physical, and emotional abuse (Gnan et al., 2019). In line with Minority Stress Theory (Meyer, 2003) these experiences underscore disproportionate mental ill health, including anxiety, depression, suicidal behaviours and self-harm, substance use, and eating disorders (e.g., Amos et al. 2020).

School climate is highly influential to LGBTQ+ students' mental health, academic performance and school attendance (Rimes et al., 2019; Johns et al., 2019; Kosciw et al., 2020; Fantus & Newman, 2021). LGBTQ+ young people exposed to racial/ethnic marginalisation may experience greater inequities within school environments that fail to regard their cultural contexts and intersectional identities (Brown et al., 2022). However, research about LGBTQ+ young people from ethnically and racially minoritised groups often reinforces deficit perspectives and perpetuates stereotypes and discrimination through an over-emphasis on mental health problems rather than normative developmental processes or positive youth development (Toomey et al., 2017). Moreover, most existing research is quantitative and US-based (Abreu et al., 2021; Newman et al., 2022) with limited UK evidence about the influence of contextual factors on effectiveness of school-based interventions to reduce mental health inequalities (McDermott et al., 2022), nor of the experiences of students with intersecting minoritised identities (Johns et al., 2019; Foulkes & Stapley, 2022).

Teachers have an important role (Poteat et al., 2021), yet many remain unaware of the challenges that LGBTQ+ students face (Harris et al., 2021) and LGBTQ+ inclusion is often implicitly the responsibility of LGBTQ+ staff (Brett, 2021). Staff professional development about sexual and gender identity has been identified as a target to improve school climate (Gower et al., 2018; Greytak et al., 2013) but this often comprises one-off, reactive sessions (Harris et al., 2021). Moreover, evidence is

limited about how to design professional development programs (Johns et al., 2019), and about barriers to uptake and implementation (McQuillan & Leininger, 2021). Grounded in an intersectional approach and coproduced with young people, this study therefore aims to understand how to create positive school environments for LGBTQ+ students and what systemic factors influence whether, and the extent to which, schools do so.

Theoretical framework

Intersectionality theory (Crenshaw, 1991) shaped the study conceptualisation and interpretation of findings. In recognising that people occupy multiple social statuses (and that this may include a mix of advantaged and disadvantaged positions), it highlights the simultaneity of intersecting forms of oppression and privilege such as white culture, cisgenderism, hetereosexism, and how these shape people's experiences. Queer theory (De Lauretis, 1991) also informed our work, particularly at analysis and interpretation stages. This describes three interrelated components relevant to our study, a) challenging heterosexuality as the norm (“heteronormativity”), b) challenging prevailing ideas that studies of sexual minorities should consider them as a single entity and c) emphasising the myriad ways in which race, class, culture, generation, geography and socio-political differences, shapes bias against sexual minority groups. Queer theory also challenges socially constructed binary assumptions and the oppressive power of dominant gender identity norms, “cisnormativity”). Drawing on these theoretical frameworks are useful in framing marginality as socioculturally created rather than an inherent pathology (Kitzie et al., 2022).

While recognising the importance of research with younger age groups, we focus on secondary/college age students (aged 13-19 years). Specifically, we address the following questions from student, staff and professional development training provider perspectives:

1. What are key components of mentally, socially and emotionally healthy school environments for LGBTQ+ students considerate of intersecting minority identities?

2. What are the information, skills and capacity needs of secondary school/college staff to support LGBTQ+ students' mental health?

3. What factors influence uptake and implementation of school initiatives to help tackle inequities experienced by LGBTQ+ students?

Methods

Design and setting

We conducted a qualitative study as part of the Schools Training to Enhance Support for LGBT+ Young People study (STEPS). This aimed to explore ways to optimise professional development for staff to improve school climate to positively influence young people's mental health. All study phases (including research question/topics, planning, ethical procedures and applications, data collection, analysis and interpretation) were co-produced by a team of young people, university researchers, and LGBTQ+ organisation representatives.

Participants and sampling

Training providers of professional development training to schools/educators about LGBTQ+-related issues. An initial scoping exercise was conducted prior to sampling and recruitment, to collect publicly available information on the number and variety of UK providers on type of training and/or resources provided, contacting some organisations for further information. To maximise variation across geographic coverage, training focus, and setting (urban/rural), we invited all identified training providers.

Staff and students were purposively sampled by advertising through the coproduction teams' networks, including LGBTQ+ organisations and secondary schools (ages 13-19 years) or colleges (in the UK these specifically serve students usually between the ages of 16-19 years), and social media. Leaflets were shared digitally with potential participants stating that anyone aged 13-19 years or

working in a school/college could participate and including positive action statements around race, ethnicity, faith and rural/coastal location. The leaflets also stated that participants did not have to identify as LGBTQ+ to participate to avoid excluding those who are questioning or not 'out'. Except for pilot focus group participants, school staff and student participants were asked to complete an optional diversity monitoring form. Training providers also did not complete the form.

Procedure

Data were collected between 21st February-28th November 2021. Potential participants indicating interest received an e-mail invitation and information including confidentiality and withdrawal details. Up to three further contacts were made. Following informed consent, participants were assigned a unique ID number and offered £15 e-gift vouchers for their time. Participants opted to participate either via online semi-structured interview or focus group and could choose for interviews either 1-to-1 with a university researcher or 2-to-1, also with a young coproduction team member. All were audio-recorded with consent. Data were transcribed verbatim, removing identifying information.

Interview/focus group guides

Topic guides were developed via literature review and discussion with the coproduction team, then piloted among the coproduction team and pilot focus group with young people from an LGBTQ+ organisation. The guide was adapted to improve clarity of wording, flow and length. Topics covered LGBTQ+ students' experiences at school/college; experiences of/with students with intersecting identities; mental health/well-being and support needs; staff information, training, and capacity needs; take up and implementation of school initiatives.

Data analysis

Data were inductively thematically analysed (Braun & Clarke, 2012). AM led on the analysis, with input and discussion throughout with the wider coproduction team who familiarised themselves with a subset of the data across the three participant groups. Across three data analysis sessions to discuss interpretations, using an online collaborative tool to capture initial codes and themes. These initial insights shaped the lead authors' interpretation of the data and following further data familiarisation, remaining transcripts were descriptively coded to develop an initial coding framework, refined through iterative discussion and coding rounds. Data were imported into NVivo (QSR International Pty Ltd, 2018) to support data management and the coding framework applied to all transcripts. Supported by discussion and visual mapping, the team sought themes reflecting relevant key patterns within and across interviews. These were continually checked and refined against transcripts, looking for patterns and for similarities/differences within and across sample groups. Themes were defined, described and labelled, and patterns and interpretations were discussed. While we are aware of the complexities around the term 'data saturation' (Braun & Clarke 2021), we appraised saturation to be the point at which additional interviews, despite their richness, were no longer leading to meaningful changes to our initial coding framework. Quotes are labelled as TP (Training Provider), YP (Young Person), or SS (School Staff). Where available, we include details of participant identified gender (labelling participants as "trans*" (as an umbrella term) to indicate where people have self-identified as 'male' or 'female' but that this was not the same as the related 'sex' assigned at birth, alongside other self-reported gender identities, to give an indication of gender diversity within sample. We acknowledge that this may not be a term the individual may use to describe themselves), sexual identity and race/ethnicity (grouped to maintain confidentiality).

Reflexivity

The co-production team consisted of individuals with various perspectives based on their lived experiences from different identities, including race and ethnicity, sexual orientation, gender diversity, and age. Due to the small team size, we agreed for specific characteristics not to be

disclosed to preserve anonymity. As a research team, we engaged in a continual and iterative practice of reflexivity. For instance, discussing the potential impacts of our positionality on research design, planning, data collection, analysis and writing.

Ethical approval

Written informed consent was gathered from all participants (for those under 16 years informed assent and parental consent was obtained). Consent/assent was re-obtained verbally (audio-recorded) at the start of interviews/focus groups. Ethical approval was received from King's College London Research Ethics Committee (Palliative care, Nursing and Midwifery Research Ethics Subcommittee), reference: HR-20/21-21515.

Results

Sample

Qualitative data were collected from 63 participants (Table 1), including 22 school and college staff, 32 students, and 9 training providers.

Table 1. Sample Characteristics of students and staff for whom diversity monitoring data were collected.

Characteristics		n	%
**Participant group**	Staff	22	47
	Students	32	53
**Region of residence**	North England	2	4
	London	38	81
	South England	7	15
**Gender identity**	Female (including trans woman)	26	55
	Male (including trans man)	11	23
	Non-binary	7	15
	Not sure/Don't know/Prefer not to say	3	6
**Gender same as assigned at birth?**	No	11	23
	Yes	36	77
**Sexual orientation**	Asexual	3	6
	Bisexual	7	15

	Gay	5	11
	Heterosexual or straight	14	30
	Lesbian	6	13
	Not sure/Don't know/Unsure or questioning/ Prefer not to say	3	6
	Pansexual	3	6
	Queer	6	13
**Ethnicity**	Asian	10	21
	Black	2	4
	Mixed/Multiple ethnic groups	2	4
	White British/ White other	30	64
	Any other group	3	6

Notes: Characteristics and samples have been grouped to preserve anonymity. Excludes training providers, and student pilot focus group participants.

We identified one overarching theme, ‘A whole school, intersectional approach which shifts the narrative’, and four themes: (1) ‘Feeling safe, seen and celebrated: embedding intersectional signs, signals and symbols’, (2) ‘Everyone’s business: and the importance of collaborative working’, (3) ‘Embedding a culture of change’, and (4) ‘Re-locating the problem: challenging deficit thinking’.

The overarching theme captured an underlying pattern discernible throughout the four themes: that creating mentally, socially and emotionally positive environments for LGBTQ+ students requires more fundamental and contextually contingent changes to schools’ structure and culture beyond staff training alone. Specifically, data supported previous findings and practice highlighting the need for a whole school approach (WSA) -meaning action across curricula, school ethos and environment, collaboration with all staff and students, as well as family and community partnerships with schools/colleges. However, participants’ accounts went further to emphasise that in enacting WSAs, an intersectional lens and actively shifting the narrative (e.g., away from problematising ‘LGBTQ+ issues’) is necessary if such approaches are to be meaningful to young people. Specifically, by recognising that inequities remain due to white, cisgender and heteronormative structural biases inherent within educational organisations, policy and society.

Feeling safe, seen and celebrated: embedding intersectional signs, signals and symbols

Many participants, across all groups, commented that the school environment and curriculum, often reflected cis-gender and heteronormativity, constraining sense of belonging for those with differing experience, inhibiting self-awareness and self-acceptance. Many interviewees described attempts to counterbalance this by enhancing the visibility of LGBTQ+ identities in the physical building (e.g., in LGBTQ+ inclusive posters, displays, flags, lanyards, gender neutral facilities), within other aspects of the school community/learning environment (e.g., within curricula, assemblies, societies, forums, clubs, within the staff body and external speakers), or through use of gender-neutral language, preferred names and pronouns.

"My old school had Pride flags along the balcony in one of the halls and it just made it seem every time you walked in there like it was a welcoming space and like the whole school reflected being a place where you felt that you were, not only like allowed to be, but celebrated for who you are. And I think those are really small things, but they can make such a huge difference in terms of how you feel." YP25, female, bisexual, white

"There are posters up throughout the school of people that anyone can talk to, including three school counsellors, on any sort of issues like that as well. So I think the school does try make it clear that there are places for people to talk about these issues and does try and to show that the school is working to improve the atmosphere for people who are, who are not kind of white, cis, het, heteronormative etc." YP22, not sure, pansexual, white

However, such experiences were not uniform and attempts at visibility commonly represented white bodies and reflected Western norms which was perceived - particularly among or on behalf of students from other backgrounds - as alienating, perpetuating the perception that LGBTQ+ issues did or should not relate to them, and/or limiting the relevance to their lived experience.

"Addressing race and intersectionality through uhm, you know when you're talking about sexuality with young people is, I guess, fundamental to helping them see it themselves, I suppose, or just helping them understand themselves." SS24, male, gay, white

"That whole thing around making intersectionality visible, uhm, making sure that if we're putting a display up about LGBT rights that we also have Black people on the display and there they are gay Muslim people, and they should know that that's acceptable. Or equally there are people who came from Polish backgrounds who are not necessarily your cis straight person and they need to know that that's OK." SS23, female, heterosexual, Asian

"Definitely representation. I think that is so important, so making it very clear that in some communities, even though it's a minority, they still believe that being LGBTQ+ is a white phenomenon. It's just a white man's issue, it's not within other communities." SS07, female, queer, Black

Another key facet of visibility, particularly identified by student and training provider participants, was representation of LGBTQ+ identities and allyship within the staff body. Their accounts suggested that seeing a diversity of identity representations within the school, would enhance feelings of belonging and self-acceptance, which in turn would positively impact upon mental health and well-being. For example, by enabling young people to express and explore themselves, challenging stereotypes about 'being LGBTQ+' and what that means or looks like, facilitating young people to be open, and to seek and access support if needed.

"I don't know any, queer POC members of staff in the college. I don't know many people like with an intersecting identity (…) At least you could feel, ok, that could be me one day." YP18, non-binary, queer, other ethnic group

"A few of my teachers wear like queer ally badges. I think that's really nice, um 'cause obviously it shows that they're a safe person to speak to, they are a safe person to just be around, I guess. Especially since I know there are staff in school who are homophobic who, it wouldn't really be a good idea for you to talk to." YP16 not sure, queer, white

Both staff and students recognised that the extent to which staff feel able to be openly out or in allyship may also be shaped by wider social and cultural forces, both experienced and anticipated, and by the extent to which homophobia is unchallenged within the school environment.

"I had an incident in which - I have like the rainbow pin on my lanyard - and a student came up to me and told me, 'oh do you support these people?' and I said 'yes' and the student went, 'well, I'm homophobic and pretty much started spitting their food and when the supervisor kind of came over [they] pretty much implied that I had invited that because I should have just not had the pin, and that problem wouldn't have appeared." SS22, female, asexual, white

Everyone's business: and the need for collaborative working

In line with the 'whole school' aspect of our overarching theme, participants across all groups recognised that staff, senior leadership and governors, young people, training providers, parents and the wider community, all have a role in creating a supportive environment, while recognising that the skills, expertise and resources needed may vary by role and context.

Most staff and training providers recognised the value of external training providers to support and facilitate such collaboration, creating spaces to share best practice, lessons learned, ask questions, and discuss challenges in a way that made staff feel safe to not know, make mistakes and learn. This was perceived to encourage staff to share experiences and engage more with training, as well as to grow in confidence in communicating around gender and sexuality.

"Making the training available to everybody, I think is really important. Um, and I think it's and having those conversations, um, between so that ideas can be shared in that collaborative practice. So, if somebody's feeling less confident in in responding or having those conversations, that they can seek advice and support from others and learn from each other." SS29, female, heterosexual, white

Participants in all groups also discussed the importance of building relationships, trust and working collaboratively with parents, carers and local communities. Building relationships (e.g., by running groups and events in local public spaces and engaging in conversations) could increase trust, awareness and understanding around sexual and gender diversity, and provide a space for transparent dialogue.

"I think what we should be doing is reaching out to our local community and putting in place spaces where the parents can come in where the community can get involved in our conversation, because ultimately, what is a school is built by the outside community. Unless we're able to help that group of people then inside school is just gonna be kind of putting bandaids everywhere." SS12, male, gay, white

"I think it's really key, as well, in school to understand the wider context, so what happens outside the classroom and what happens within. Uhm, and understanding certain barriers, dynamics, uhm, what's happening in social contexts, economic contexts, specific areas, or within the college as a whole but also out in the communities. And understanding how, uhm, that might affect how people perceive and experience." SS29, female, heterosexual, white

In this, co-designing professional development training with schools and staff to tailor content to the local context was commonly identified by staff and training providers as key; particularly to develop skills to speak more confidently in culturally sensitive ways and to engage in 'difficult' conversations

where beliefs or opinions about sexuality and gender may differ and/or within cultures where there is more stigma and less familiarity with LGBTQ+ identities.

"Just learning about the LGBT community makes some parents really uncomfortable, so I'm not sure how the training would like go with them. It probably would be negative for them, but yeah…I guess a suggestion would be like maybe do like an information evening and explaining, why it's still important for their children to learn about this, these type of issues." YP13, female (trans*), lesbian, Black

"I think maybe that would be quite useful, having, how to tackle difficult parents. What do you say to a parent who is so against anything LGBTQ+? Like you can't teach my child that no, they can't go to that lesson. So it might be worth that how to have these difficult conversations with parents, what do we say?" SS08, female, heterosexual, other ethnic group

However, it was also recognised that assuming particular parent groups/local communities were inherently less accepting or knowledgeable of LGBTQ+ identities could serve to perpetuate prejudicial views and exacerbate difference.

"I don't want to say anything that, sounds like it's deficit ideology around what other cultures and communities might feel, so I'm not going to. Uhm, I just know that everyone's journey is different and the fact that we have less young people of colour tell us their orientation, sexual orientation or their feelings around gender, will have other factors and I'm gonna be honest with you and say that I personally don't wanna make any assumptions." SS05, female, heterosexual, Asian

Collaboration with students was also included within this theme, emphasising the need to ensure their perspective, experience and voices are included and valued in school initiatives, and to facilitate peer-to-peer support. Linked to the intersectional perspective, student participants

highlighted the importance of engaging with a broad range of young people from different social groups.

"Actually listening to young people, because I feel as if with adults, I do it myself at work and at school, we feel like we know the information, we feel like we've been there, don't worry, just listen to me. But listening to the young people and finding out exactly what they need and what they want." SS07, female, queer, Black

"*Things that work well for one, for one group of people, won't necessarily work well for another group of people. So, I think it's a case if it's speaking to like, ethnic minority, like Black and ethnic minority groups, and other faith groups and like, you know, people who live in rural areas*." YP14, non-binary, bisexual, white

Embedding a culture of change

This theme highlighted the importance of sustained, consistent actions which could help actively unpick norms engrained within the school system. For example, participants across all groups talked about the importance of sustained LGBTQ+ representation – i.e., not just during Pride month; avoiding one off, tick box training and development activities; embedding diverse LGBTQ+ identities appropriately into the curriculum across all lessons, rather than just when discussing 'LGBTQ+ issues'; and, importantly, consistency in responding to homo/bi/transphobic bullying.

"I think constant, constant training and constant education. Um, it's something like I've I was quite naive. I thought that doing LGBT month will open up everybody's eyes and it'll be a situation where homophobia will disappear in a school and transphobia will disappear in school, but that's not the case. It has to be done every, it has to be constant drip feed." SS07, female, queer, Black

"Being consistent in challenging homophobic biphobic and transphobic behaviour within the schools, that every single staff member is you know challenging it. It's not just that one staff member with who gets given the rainbow lanyard and you know that responsibility." TP05

Such consistency was perceived to avoid actions being perceived as tokenistic, unauthentic and performative, and to encourage sustained iterative learning and development among staff and students to support more meaningful change. It also therefore linked to 'shifting the narrative', moving away from conceptualising intersectional 'LGBTQ+ issues' as something separate, perceived to contribute to 'othering'.

"If you're LGBT for example, you're not just LGBT for pride month, your LGBT for the whole whole year(…)So, there is an initiative at the moment where we're trying to plan in points in the curriculum to, to introduce things in a normalised way rather than as a tokenistic way, I suppose." SS28, male, gay, white

However, across groups, participant's accounts indicated that meaningful change also depends upon more fundamental shifts beyond interpersonal behaviours. For instance, proactively embedding intersectionality within initial teacher training, and making relevant school/college values, ethos, and policies explicitly visible to staff, students and parents/communities.

"What it isn't, is doing the blanket LGBT awareness training, ticking the box and moving on. From our perspective, it's about you know how do we actually, you know, it's about that structure of pastoral care, the ethos of the school community, your policies and your curriculum, is I think that the most effective way of actually embedding a culture of change." TP04

Here, factors such as leadership support, school type (e.g., faith vs. secular, private vs. comprehensive) and social, economic and cultural context (e.g., rural versus inner city, local

demographic profile) was seen to shape the extent to which and how schools had the capacity or will to engage with LGBTQ+ inclusion consistently.

"I think especially since I'm in a faith school, there's just a lot of reluctancy to move with the times. It's all about being traditional, any sort of liberal step forward they take is met with resistance from, you know, the governors and parents and everything that they teach in school has to be approved by the diocese." YP16, not sure, queer, white

"There's those schools are rich in the terms, in terms of money and might be a state school, but demographically, they've got largely middle class, mostly white pupils, with parents who are educated, professional people, who aren't going to come charging up and they will buy it. And then the opposite end, the schools which are really poor with time and poor with resources and they've got such a lot of issues going on. So they might have quite a high BAME proportion of pupils on roll. They are the schools that are being targeted by the government around Prevent, FGM [female genital mutilation], all of those issues are coming their way. So this [LGBTQ+] issue sits in a suite of issues that they've got to deal with, because they've got, you know, a cohort of pupils within the school that are homophobic and that might be coming from culture, but they need to do something about that." TP01

Re-locating the problem: challenging deficit thinking

Pertinent to the shifting the narrative aspect of our overarching theme, this last theme reflects a perceived need to move away from perceptions which problematise LGBTQ+ young persons', challenge deficit thinking and relocate the 'problem' with cis and heteronormativity. Participants across groups discussed an over-emphasis on pathologising discussions about LGBTQ+ students. This was perpetuated in an everyday sense through slurs about gender diversity and sexual orientation often used among students to connote negative opinions (e.g., "that's so gay"). Another example given by several students and training providers was of staff erroneously viewing a young person

‘coming out’ as a safeguarding issue requiring them to break confidentiality to family members. This was a barrier to students’ accessing support but could also put them at risk if such involuntary ‘outing’ leads to familial/community rejection. Such a scenario could occur for any young person but participants particularly discussed this issue in relation to students from (or perceived to be from) cultural backgrounds less aware of, open to or supportive of sexual and gender diversity and reflected a lack of cultural sensitivity and awareness within schools.

I used to go to quite like a very, very sort of like attempting to be secular school. Where like they would just like, try as hard they could to not sort of push like any kind of religion(…)in an attempt to be secular, they kind of like, get blinded to relevant considerations about people's religion, and I think that can put people at more of a risk of being outed to potentially unsupportive families. YP15, non-binary, bisexual, white

“One of the teachers actually, caused them to nearly be made homeless, because a few, more than one student was made to come out. But this particular student came from a Muslim household, and the parents were very not ok with it(….)the teacher made them come out and they were nearly kicked out their house and their church, like their mosque, yeah, because they were trans.” YP focus group participant

Such deficit-based assumptions may be exacerbated if staff members themselves hold beliefs that run counter to inclusive practice, and/or who are less familiar with concepts around gender and sexual identity.

“Older teachers who, um, or staff members who, just the way that they've been brought up has been, with this with these type of issues being kind of more swept under the carpet. And so they might, just because that's been instilled in them, they might find it quite uncomfortable to have these types of conversations, and also maybe might not know kind of what's right and wrong in today's world as

much as teachers that are younger know it. And maybe as well teachers from different cultures where it is less talked about too, or less socially acceptable." YP25, female, bisexual, white

Deficit thinking was particularly discussed in relation to gender identities due to limited staff understanding, wider societal transphobic discourse and perceptions that trans* people need to be specially accommodated (e.g., through pronouns, toilets etc). This problematises the young person and burdens them, and also reflects resistance to or lack of insight into the need to challenge underpinning norms around gender embedded within and beyond schools.

"I know about a lot of schools that aren't progressive, who just like view sort of, like queer students, particularly like a lot of trans students I know as kind of more of a logistical problem than, like you know, treating them like a human being who deserves like, help from them. They're viewed as more of a like a sort of, you know, like puzzle, that they have to solve, in terms of their place in the school." YP15, female (trans*), heterosexual, white

Participants across groups commonly raised that discussing, educating, and supporting people in relation to sexual orientation and gender identity is often seen as LGBTQ+ staff and students' 'problem' and responsibility, risking burnout.

"It's particularly acute for those in the trans community, rather than the wider community because I think they've also had a burden of like feeling they need to explain to their peers as well. And like educate their peers at the same time as trying to fight, figure out who they are and who they want to be. Um, and they're kind of, the trans community within the school are also the ones who kind of are the first to have a change for uniform and the first to have a change, for toilets." SS25, male, heterosexual, white

Discussion

We aimed to understand student, staff and training provider perspectives on how school environments could be enhanced for LGBTQ+ students while considering intersecting minoritised identities; information, skills and capacity needs of staff; and factors influencing uptake and implementation. One overarching theme captured the need for an intersectionality-informed, contextually adaptable, whole school approach (WSA) which ‘shifts the narrative’ away from deficit thinking and challenges prevailing white, cis/heteronormative norms. Underpinning this were four themes: (1) ‘Feeling safe, seen and celebrated: embedding intersectional signs, signals and symbols’, (2) ‘Everyone’s business: the need for collaborative working’, (3) ‘Embedding a culture of change’, and (4) ‘Re-locating the problem: challenging deficit thinking’.

Comparison with prior research

Our findings support the need for fundamental and proactive shifts in school culture and ethos to ensure they are positive environments for LGBTQ+, particularly gender diverse, students (e.g., Bower-Brown et al., 2021). WSAs have been previously advocated for by practitioners in this area (Dellenty, 2019) and in recent research exploring how school climate influences LGBTQ+ student mental health (McDermott et al., 2023). Although multiple conceptualisations of WSAs exist (e.g., to anti-bulling, socio-emotional learning), our findings fit with common core principles such as the importance of students, staff, families and external stakeholders working together to embed inclusive practice within and outside of school (Brown et al., 2021). This includes anti-bullying policies with specific regard for LGBTQ+ students (Hall, 2017), support spaces, as well as curriculum and staff representation of intersectional LGBTQ+ identities (e.g., Dellenty, 2019). A meta-analysis (Goldberg et al., 2019) found that WSAs to enhancing student social and emotional development showed small but significant improvements to student outcomes. Supporting our findings, they report that interventions incorporating collaborative working with the wider community including engagement with parents and local agencies were identified as more effective than those which do not. Indeed, family and peer support, access to counselling services and having trusted adults at

school have been found to be protective of depression and suicide-related behaviours among LGBTQ+ students (Rivas-Koehl et al., 2021).

Staff and students perceived lack of support, knowledge, confidence and skills to constrain communication about LGBTQ+ issues in faith schools and in contexts where a large proportion of the student body are from cultural backgrounds perceived to be less accepting of sexual and gender diversity. While some evidence explores this in relation to Catholic schools (McShane & Farren, 2023), further work is needed to unpick these concerns in greater detail to avoid generalising interpretations and perpetuating stereotypes. Nonetheless our study supports an urgent need for school LGBTQ+ initiatives to embed intersectional awareness and shifting narratives away from deficit-perspectives which problematise LGBTQ+ experiences and identities. Not least, this is key to ensure students receive support and education which is culturally sensitive, relevant, and safe (Aguilar-Hernández, 2020). Moreover, that approaches go beyond individualised and tick-box approaches involving learning 'about' difference that perpetuate othering of sexual and gender minorities, particularly those with intersecting marginalised statuses (Curtis et al., 2019) and those from faith communities (McShane & Farren, 2023). Instead, approaches which critically appraise ways in which power and oppression are inherent within cis/heteronormative and white, Eurocentric systems are needed (Curtis et al., 2019).

Increasing numbers of young people self-identify as trans*, the 'debates' around gender and trans* rights are often transphobic, questioning the validity of trans* identity, and ignoring the substantial health and social inequities they face (Pearce et al., 2020; McDowell, 2021). Bower-Brown et al. (2020), suggest that the "British school system is fundamentally unsuitable for non-binary and gender-questioning identities" (p74). Our findings support this and other UK-based evidence that trans* young people may experience particular difficulties with unsupportive school climates and/or

lack of staff knowledge, and that this is influenced by cis/heteronormativity and wider discourse (Horton, 2023).

In line with prior research (e.g., Ioverno et al., 2022; McDermott et al., 2023), our findings advocate for mandatory and continuous staff professional development as a key component of any WSA. Aligned with prior research, we identified barriers to implementing /enacting LGBTQ+ affirmative initiatives such as: lack of confidence and knowledge, confusion around laws and policies and fears of parental response (Meyer et al., 2019), alongside lack of government support and guidance (Glazzard & Stones, 2021). Our study adds to this by acknowledging differences in support for, and inequities in the capacity of, schools to implementing change linked to governance (e.g., faith schools) and socio-demographic constraints. Such differences are likely to create a widening of inequities experienced by LGBTQ+ students, particularly those also minoritised on the basis of religion, race, socio-economic resources, and those from cultural contexts less likely to be familiar or accepting of gender and sexual diversity. It is important to acknowledge that any shift away from intolerance is beneficial and that schools progress at different paces, thus change is required at multiple levels (Shattuck et al., 2021).

Strengths and limitations

A strength of our study is the inclusion of multiple perspectives. While research about young people's experiences rightly should foreground their voice, it should also balance this with the need to shift responsibility from young people to those with a duty to care for them. In practice it is staff and trainers tasked with enacting change (whether passed through legislation, inter or intra-personally mediated) and thus understanding their take (while being critically aware of how their positionality shapes their viewpoints) is essential. Moreover, in coproducing our study with a team of young people we ensured that direct lived experience was foregrounded in the design, analysis and interpretation. A limitation was that we did not include the perspectives of parents/carers

which, given their core role in WSAs, is an important avenue for future work. Last, while our sample overall included diversity in relation to faith, ethnicity, gender and sexual orientation, white British people comprised 56% of the staff and 60% of the student sample. It is important to critically consider this in relation to their perspectives on the experiences of students with intersecting minoritised identities and in our interpretations of this.

Conclusion

Our findings support calls for WSAs to LGBTQ+ inclusive practice, going further to impress the need to embed intersectional experience and awareness from the outset. In doing so, we emphasise the need to more fundamentally 'shift the narrative', away from deficit thinking towards challenging complicit cis/heteronormative and White education structures. However, there is a risk of widening inequities, particularly for LGBTQ+ young people with intersecting minoritised identities as the capacity to embed such practices are affected by school wealth, parent pressures, and socio-cultural context. Further research is needed on approaches to collaboration with a diverse range of parents and communities, and to develop evidence-based implementation strategies to support uptake and adoption of inclusive practices.

Acknowledgments

We would like to thank River Újhadbor for their support with workshops and advice related to the study.

This work was supported by the TRIUMPH (Transdisciplinary Research for the Improvement of Youth Mental Public Health) Network which is funded by the Cross-Disciplinary Mental Health Network Plus initiative supported by UKRI under grant ES/S004351/1. AM, CW, and GK are part supported by the ESRC Centre for Society and Mental Health at King's College London [ES/S012567/1]. The views expressed are those of the author(s) and not necessarily those of the ESRC or King's College London.

References

Abreu RL, Audette L, Mitchell YL, Simpson I, Ward J, Ackerman L, Gonzalez KA, Washington K. LGBTQ student experiences in schools from 2009–2019: A systematic review of study characteristics and recommendations for prevention and intervention in school psychology journals. Psychology in the Schools. 2022 Jan;59(1):115-51.

Aguilar-Hernández JM. Queering critical race pedagogy: Reflections of disrupting erasure while centering intersectionality. International Journal of Qualitative Studies in Education. 2020 Jul 2;33(6):679-94.

Amos R, Manalastas EJ, White R, Bos H, Patalay P. Mental health, social adversity, and health-related outcomes in sexual minority adolescents: a contemporary national cohort study. The Lancet Child & Adolescent Health. 2020 Jan 1;4(1):36-45.

Argyriou A, Goldsmith KA, Tsokos A, Rimes KA. Psychosocial mediators of the relations between sexual orientation and depressive symptoms in a longitudinal sample of young people. Psychology of Sexual Orientation and Gender Diversity. 2020 Jun;7(2):142.

Bower-Brown S, Zadeh S, Jadva V. Binary-trans, non-binary and gender-questioning adolescents' experiences in UK schools. Journal of LGBT Youth. 2023 Jan 2;20(1):74-92.

Braun V, Clarke V. To saturate or not to saturate? Questioning data saturation as a useful concept for thematic analysis and sample-size rationales. Qualitative Research in Sport, Exercise and Health. 2021 Mar 4;13(2):201-16.

Braun V, Clarke V. Using thematic analysis in psychology. Qualitative Research in Psychology. 2006 Jan 1;3(2):77-101.

Brett A. Changing the narrative. A photo elicitation study of LGBT+ secondary school teachers in England (Doctoral dissertation, Nottingham Trent University), 2021.

Brown JM, Naser SC, Brown Griffin C, Grapin SL, Proctor SL. A multicultural, gender, and sexually diverse affirming school-based consultation framework. Psychology in the Schools. 2022 Jan;59(1):14-33.

Brown R, Van Godwin J, Edwards A, Burdon M, Moore G. Development of a theory of change and evaluability assessment for the whole school approach to mental health and emotional wellbeing. Available: https://gov.wales/whole-school-approach-mental-and-emotional-wellbeingevaluability-assessment

Crenshaw K. Mapping the Margins: Intersectionality, Identity Politics, and Violence Against Women of Color. Stanford Law Review. 1991 Jul;43(6):1241-99.

Curtis E, Jones R, Tipene-Leach D, Walker C, Loring B, Paine SJ, Reid P. Why cultural safety rather than cultural competency is required to achieve health equity: a literature review and recommended definition. International Journal for Equity in Health. 2019 Dec;18(1):1-7.

De Lauretis T. Queer theory: Lesbian and gay sexualities an introduction. Differences. 1991 Jul 1;3(2):iii-xviii.

Dellenty S. Celebrating Difference: A whole-school approach to LGBT+ inclusion. Bloomsbury Publishing; 2019 May 30.

Fantus S, Newman PA. Promoting a positive school climate for sexual and gender minority youth through a systems approach: A theory-informed qualitative study. American Journal of Orthopsychiatry. 2021;91(1):9.

Fisher E, Anhalt K, Cannava A, Gorenstein S, Saint J, Schanding Jr T, Tiberi AE, Varjas K. Safe and Supportive Schools for LGBTQ+ Youth, National Association of School Psychologists, 2017.

Foulkes L, Stapley E. Want to improve school mental health interventions? Ask young people what they actually think. Journal of Philosophy of Education. 2022 Feb;56(1):41-50.

Glazzard J, Stones S. Running Scared? A Critical Analysis of LGBTQ+ Inclusion Policy in Schools. Frontiers in Sociology. 2021:64.

Gnan GH, Rahman Q, Ussher G, Baker D, West E, Rimes KA. General and LGBTQ-specific factors associated with mental health and suicide risk among LGBTQ students. Journal of Youth Studies. 2019 Nov 26;22(10):1393-408.

Goldberg JM, Sklad M, Elfrink TR, Schreurs KM, Bohlmeijer ET, Clarke AM. Effectiveness of interventions adopting a whole school approach to enhancing social and emotional development: a meta-analysis. European Journal of Psychology of Education. 2019 Oct 15;34:755-82.

Gower AL, Forster M, Gloppen K, Johnson AZ, Eisenberg ME, Connett JE, Borowsky IW. School practices to foster LGBT-supportive climate: Associations with adolescent bullying involvement. Prevention Science. 2018 Aug;19:813-21.

Greytak EA, Kosciw JG, Boesen MJ. Educating the educator: Creating supportive school personnel through professional development. Journal of School Violence. 2013 Jan 1;12(1):80-97.

Hall W. The effectiveness of policy interventions for school bullying: A systematic review. Journal of the Society for Social Work and Research. 2017 Mar 1;8(1):45-69.

Harris R, Wilson-Daily AE, Fuller G. Exploring the secondary school experience of LGBT+ youth: an examination of school culture and school climate as understood by teachers and experienced by LGBT+ students. Intercultural Education. 2021 Jul 4;32(4):368-85.

Horton C. Institutional cisnormativity and educational injustice: Trans children's experiences in primary and early secondary education in the UK. British Journal of Educational Psychology. 2023 Mar;93(1):73-90.

Johns MM, Poteat VP, Horn SS, Kosciw J. Strengthening our schools to promote resilience and health among LGBTQ youth: Emerging evidence and research priorities from The State of LGBTQ Youth Health and Wellbeing Symposium. LGBT health. 2019 Jun 1;6(4):146-55.

Kitzie, V.L., Wagner, T.L., Lookingbill, V. and Vera, N., 2022. Advancing information practices theoretical discourses centered on marginality, community, and embodiment: Learning from the experiences of lesbian, gay, bisexual, transgender, queer, intersex, and asexual (LGBTQIA+) communities. Journal of the Association for Information Science and Technology, 73(4), pp.494-510.

Kosciw JG, Clark CM, Truong NL, Zongrone AD. The 2019 National School Climate Survey: The Experiences of Lesbian, Gay, Bisexual, Transgender, and Queer Youth in Our Nation's Schools. A

Report from GLSEN. Gay, Lesbian and Straight Education Network (GLSEN). 121 West 27th Street Suite 804, New York, NY 10001; 2020.

Ioverno S, Bishop MD, Russell ST. Does a decade of school administrator support for educator training on students' sexual and gender identity make a difference for students' victimization and perceptions of school climate? Prevention Science. 2022 Jan 1:1-1.

McDermott E, Kaley A, Kaner E, Limmer M, McGovern R, McNulty F, Nelson R, Geijer-Simpson E, Spencer L. Reducing LGBTQ+ adolescent mental health inequalities: A realist review of school-based interventions. Journal of Mental Health. 2023 Aug 17:1-1.

McDermott E, Kaley A, Kaner E, Limmer M, McGovern R, McNulty F, Nelson R, Geijer-Simpson E, Spencer L. Understanding how school-based interventions can tackle LGBTQ+ youth mental health inequality: a realist approach. International Journal of Environmental Research and Public Health. 2023 Feb 28;20(5):4274.

McDowell KE. Trans-formational? Why we need to do better for trans young people in the UK. The British Student Doctor Journal. 2021 Apr 30;5(2):75-84.

McQuillan MT, Leininger J. Supporting gender-inclusive schools: Educators' beliefs about gender diversity training and implementation plans. Professional Development in Education. 2021 Jan 1;47(1):156-76.

McShane S, Farren M. An Exploration of Teachers' Knowledge and Awareness of Issues and Barriers Encountered in Supporting LGBT+ Students: A Study in a Catholic Secondary School in Ireland. Equity & Excellence in Education. 2023 Apr 3;56(1-2):114-28.

Meyer EJ, Quantz M, Taylor C, Peter T. Elementary teachers' experiences with LGBTQ-inclusive education: Addressing fears with knowledge to improve confidence and practices. Theory Into Practice. 2019 Jan 2;58(1):6-17.

Meyer IH. Prejudice, social stress, and mental health in lesbian, gay, and bisexual populations: conceptual issues and research evidence. Psychological Bulletin. 2003 Sep;129(5):674.

Newman PA, Reid L, Lacombe-Duncan A, Tepjan S, Ramsundarsingh S. A methodological scoping review of qualitative research on LGBTQ+ bullying victimization: Implications for diversity, equity, and inclusion. Journal of Gay & Lesbian Social Services. 2022 Oct 2;34(4):521-55.

Pearce R, Erikainen S, Vincent B. Afterword: TERF wars in the time of COVID-19. The Sociological Review. 2020 Jul;68(4):882-8.

Poteat VP, Watson RJ, Fish JN. Teacher support moderates associations among sexual orientation identity outness, victimization, and academic performance among LGBQ+ youth. Journal of youth and adolescence. 2021 Aug;50(8):1634-48.

QSR international Pty ltd. NVivo qualitative data analysis software. Version 10: QSR international Pty ltd, 2010.

Rimes KA, Shivakumar S, Ussher G, Baker D, Rahman Q, West E. Psychosocial factors associated with suicide attempts, ideation, and future risk in lesbian, gay, and bisexual youth. Crisis. 2018 Jun 21.

Rivas-Koehl M, Valido A, Espelage DL, Robinson LE, Hong JS, Kuehl T, Mintz S, Wyman PA. Understanding protective factors for suicidality and depression among US sexual and gender minority adolescents: Implications for school psychologists. School psychology review. 2022 Jun 3;51(3):290-303.

Russell ST, Fish JN. Mental health in lesbian, gay, bisexual, and transgender (LGBT) youth. Annual Review of Clinical Psychology. 2016 Mar 28;12:465-87.

Shattuck D, Hall JL, Dickson E, Green AE, Ramos MM. Implementing evidence-informed practices to make schools safer and more supportive of sexual and gender minority youth. The Journal of school health. 2021 Mar;91(3):258.

Taylor A, McMellon C, French T, MacLachlan A, Evans R, Lewis R, McCann M, Moore L, Murphy S, Simpson S, Inchley J. Defining research priorities for youth public mental health: reflections on a coproduction approach to transdisciplinary working. Health Research Policy and Systems. 2022 Jun 20;20(1):72.

Toomey RB, Huynh VW, Jones SK, Lee S, Revels-Macalinao M. Sexual minority youth of color: A content analysis and critical review of the literature. Journal of gay & lesbian mental health. 2017 Jan 2;21(1):3-1.

Ethical approval letter

Click here to access/download
Supporting Information
Ethics Decision Letter_fully approved 060121.pdf
