# Supplementary material for: Intersectionality informed and narrative-shifting whole school approaches for LGBTQ+ secondary school student mental health: A UK qualitative study
Source: PLoS One. 2024 Jul 11;19(7):e0306864. doi: 10.1371/journal.pone.0306864 (PMC11238970; doi:10.1371/journal.pone.0306864)
Supplement: Attachment — Submitted filename: Response to Reviewers.docx [file pone.0306864.s003.docx]

Dear reviewers,

Thank you for your considered, helpful and clear comments on our paper entitled, “Intersectionality informed and narrative-shifting whole school approaches for LGBTQ+ secondary school student mental health: A UK qualitative study”. We have made tracked changes in the revised document in response to the comments on grammar and clarification of terms (e.g., ‘young people’), as well as on content. We focus on the content comments here for clarity.

1. More information about coproduction as an approach.

Thank you, we agree and have added a definition of coproduction, as well as brief rationale for taking this approach in research with young people (p.5 lines 17-21)

2. **Add %/numbers of how many people expressed which topics for reference.** Thank you for this suggestion which we do understand. However, we have chosen not to report percentages as it suggests a more positivist approach than that taken. This may have been appropriate if we had used an approach such as content analysis but we consider it incompatible with the reflexive thematic analytic approach we took. For example, see for a discussion:

Neale, J., Miller, P., & West, R. (2014). Reporting quantitative information in qualitative research: guidance for authors and reviewers. Addiction, 109, 175–176. Available: <https://onlinelibrary.wiley.com/doi/pdf/10.1111/add.12408>

3. **Page 14, line 2. “could add more context here about cultural differences in school settings, parent-teacher/staff interactions, and WSAs' approach to these discussions.”** We very much agree that these points merit more detailed and nuanced discussion. For the purposes of this paper and the need to balance succinctness in word count with detail, we decided to take an approach which provided more of an overarching perspective. We aim to offer a second paper which delves more deeply into context, parent/teacher interactions and WSA approaches to community and parental engagement. To acknowledge this, we have added a line into the discussion (p.21 lines 10-12)
4. **On terminology (female’/male vs woman/man)** we agree, thank you. This initially reflected how the diversity monitoring form was structured but agree that it is inappropriate, so have amended to ‘woman’ and ‘man’ throughout.
5. **Clarify ‘deficit thinking’.** Thank you we have added a reference and description (p.18 lines 1-2)

In addition, we note and appreciate the additional comments provided by the Editor. Please find below our responses to these:

6. **I believe that the introduction and theoretical framework sections could benefit from more citation and discussion of previous research involving intersectionality in LGBTQ+ youth.**

Thank you, we were mindful of the length of submission and of providing a background that would speak to research about school experiences in particular. We have gladly added the following references which together provide a broader perspective, while acknowledging the limitations in scope to offer a comprehensive review of the literature.

Moffitt U, Juang LP, Syed M. Intersectionality and youth identity development research in Europe. Frontiers in psychology. 2020 Jan 31;11:78.

Huang YT, Ma YT, Craig SL, Wong DF, Forth MW. How intersectional are mental health interventions for sexual minority people? A systematic review. LGBT health. 2020 Jul 1;7(5):220-36.

Kim SE, Toomey RB, Anhalt K. Latinx sexual minority youth's identity development and experiences with preparation for bias. Family Relations. 2023 72(3): 948-65.

7. **Were the authors specifically interested in particular types of intersectionality as they relate to LGBTQ+ youth? (e.g., race/ethnicity, class, rurality/urbanicity, etc)?**

Thank you for this clarification. Our study predominantly focused on intersections between race/ethnicity (and/or) religion/faith and LGBTQ+ status. While we originally intended to explore how these intersections may be differentially experienced in urban and more rural areas, in practice our sample was predominantly urban. We have added clarification in the results (p.8 line 24).

8. **More detail on the content of the interview guide would be helpful--the authors should also consider including this as an appendix in the manuscript.**

Thank you, we agree this would provide important detail and context to the results. We therefore have added further detail in the methods section including example questions from each topic guide (p.7 lines 7-20), as well as submitted the topic guide as an appendix.
