# Supplementary material for: Intersectionality informed and narrative-shifting whole school approaches for LGBTQ+ secondary school student mental health: A UK qualitative study
Source: PLoS One. 2024 Jul 11;19(7):e0306864. doi: 10.1371/journal.pone.0306864 (PMC11238970; doi:10.1371/journal.pone.0306864)
Supplement: Attachment — Submitted filename: Response to Reviewers.docx [file pone.0306864.s005.docx]

Thank you for your feedback on the revisions made to our paper, ‘Intersectionality informed and narrative-shifting whole school approaches for LGBTQ+ secondary school student mental health: A UK qualitative study.” (Reference PONE-D-23-28894R1). Please find below a point-by-point response to the issues raised in your e-mail.

The authors are advised to conduct a thorough revision of the manuscript to improve readability

**Thank you, we have re-read the manuscript to appraise readability and note that we have responded to and revised all suggestions for wording, grammar and phrasing made by the reviewer. We are confident that the manuscript is readable as it stands.**

Please review your reference list to ensure that it is complete and correct. If you have cited papers that have been retracted, please include the rationale for doing so in the manuscript text, or remove these references and replace them with relevant current references. Any changes to the reference list should be mentioned in the rebuttal letter that accompanies your revised manuscript. If you need to cite a retracted article, indicate the article’s retracted status in the References list and also include a citation and full reference for the retraction notice

**Thank you, we have checked our references and there are no retracted references. We have made several revisions to the referencing format after checking against the PLOS ONE reference guide to ensure they are all complete and correct.**

Reviewer #1: The authors have done an excellent job addressing the majority of the issues brought up and created a stronger, more organized manuscript. The minor revisions in the document attached zero in on grammar errors (missing comments, run-on sentences, missing words/phrases). After these minor changes are made, the document should be ready for publication.

Excellent work!

**Thank you for your helpful feedback on the manuscript and revisions – we are grateful for your comments and have made those changes suggested in the revised manuscript.**
